# Transcriptomic analysis of grain filling in rice inferior grains under moderate soil drying

**DOI:** 10.1093/jxb/erz010

**Published:** 2019-01-23

**Authors:** Guan-Qun Wang, Hao-Xuan Li, Lei Feng, Mo-Xian Chen, Shuan Meng, Neng-Hui Ye, Jianhua Zhang

**Affiliations:** 1 Southern Regional Collaborative Innovation Center for Grain and Oil Crops in China, Hunan Agricultural University, Changsha, China; 2 Department of Biology, Hong Kong Baptist University, Kowloon, Hong Kong; 3 School of Life Sciences and State Key Laboratory of Agrobiotechnology, The Chinese University of Hong Kong, Shatin, Hong Kong; 4 Shenzhen Research Institute, The Chinese University of Hong Kong, Shenzhen, China

**Keywords:** Abscisic acid, grain filling, inferior grains, *Oryza sativa*, RNA sequencing, soil drying

## Abstract

Moderate soil drying imposed at the post-anthesis stage significantly increases starch accumulation in inferior grains of rice, but how this process is regulated at the level of gene expression remains unclear. In this study, we applied moderate drying (MD) treatments to the soil at the post-anthesis stage and followed the dynamics of the conversion process of soluble sugars to starch in inferior grains using RNA-seq analysis. An elevated level of ABA induced by MD was consistently associated with down-regulation of *ABA8ox2*, suggesting that lower expression of this gene may be responsible for the higher ABA content, potentially resulting in better filling in inferior grains. In addition, MD treatments up-regulated genes encoding five key enzymes involved sucrose-to-starch conversion and increased the activities of enzymes responsible for soluble-sugar reduction and starch accumulation in inferior grains. Differentially expressed transcription factors, including NAC, GATA, WRKY, and M-type MADS, were predicted to interact with other proteins in mediating filling of inferior grains as a response to MD. Transient expression analysis showed that NAC activated *WAXY* expression by binding to its promoter, indicating that NAC played a key role in starch synthesis of inferior grains under MD treatment. Our results provide new insights into the molecular mechanisms that regulate grain filling in inferior grains of rice under moderate soil drying.

## Teaser

Carbohydrate accumulation in inferior grains in rice panicles subjected to moderate post-anthesis water stress is regulated by NAC transcription factors that activate the expression of *WAXY*.

## Introduction

Rice panicles are composed of many spikelets, each of which is considered as an individual unit in the inflorescence (Mohapatra and Sahu, 1991). Grain filling is the process by which fertilized ovaries in each of the spikelets develop into caryopses with accumulated starch in the kernel. The structural units of rice inflorescences include the rachis, ciliate ring, and peduncle, where primary branches grow on the rachis, and secondary branches grow on the first branches. Floret inserts growing on both the primary and secondary branches are referred to as spikelets. The grain filling of spikelets is based on the order within a single panicle. Spikelets located on apical primary branches usually flower earlier and generate larger and heavier grains, referred to as ‘superior grains.’ In contrast, some spikelets located on the proximal secondary branches reach anthesis later and generate smaller or even no grains; consequently, these are referred to as ‘inferior grains’ (Mohapatra *et al.*, 1993). The differences observed between superior and inferior grains, such as ethylene production and the grain-filling rate, are more significant in large-panicle rice, especially in so-called ‘super rice’ varieties, which possess numerous spikelets with a large sink capacity and hence have a high yield potential (Panda *et al.*, 2009; Sekhar *et al.*, 2015). However, inferior spikelets usually fail to fill completely due to a slow grain filling rate, which results in only partially filled grains (Peng *et al.*, 1999; Ao *et al.*, 2008; Yang and Zhang, 2010). Grain filling mainly consists of the transportation of soluble sugars from source tissues to the spikelets, followed by starch synthesis and accumulation. Starch accumulation in grains is therefore of great importance, as it is the major constituent of the seeds (Yoshida *et al.*, 1976).

In addition to carbohydrate supply as a limiting factor, a small sink capacity, imbalances in endogenous plant hormones (Yang *et al.*, 2002, 2008), and low activities of enzymes related to sucrose and starch metabolism may all lead to a slow grain-filling rate and only partially filled inferior grains. Grain filling is a complex process involving many biochemical steps that require a large number of enzymes (Nakamura *et al.*, 1989). Among the major enzymes involved, four are considered to be key in this process: sucrose synthase (SuSase; EC 2.4.1.13), adenosine diphosphate glucose pyrophosphorylase (AGPase; EC 2.7.7.27), starch synthase (StSase; EC 2.4.1.21), and the starch branching enzyme (SBE; EC 2.4.1.18) (Nakamura *et al.*, 1989; Zhang *et al.*, 2012). The poor grain filling of inferior grains has been suggested to be attributable to the low activity of crucial enzymes participating in sucrose-to-starch conversion within the grains (Nakamura and Yuki, 1992; Kato *et al.*, 2007; Mohapatra *et al.*, 2011). In rice crop production, moderate soil water stress, particularly when imposed during the middle- to late-stages of grain filling, has been shown to improve grain yield dramatically by promoting the remobilization of carbon reserves from the stems to grains (Yang and Zhang, 2006). Furthermore, the enzyme activities of SuSase, soluble and insoluble invertase, SBE, and soluble starch synthase (SSS) are enhanced by soil drying (Zhang *et al.*, 2012).

The plant hormones that mediate spikelet development, especially ethylene and abscisic acid (ABA), play important roles in regulating grain filling. Application of ethylene inhibitors improves the dry matter partitioning and the development of inferior grains by enhancing the expression of genes encoding starch-synthesizing enzymes and endosperm cell-cycle regulators (Mohapatra *et al.*, 2000; Panda *et al.*, 2016). A higher rate of ethylene evolution in developing seeds suppresses the expression of most starch-synthesis genes and inhibits the activities of starch synthesis-related enzymes, and thus leads to a low grain-filling rate (Yang *et al.*, 2006*a*; Zhu *et al.*, 2011; Panda *et al.*, 2015). *ETHYLENE RESPONSE2* (*ETR2*), an ethylene-receptor gene, delays flowering and causes starch accumulation in stems that is not translocated to grains, leading to low grain weight in rice (Wuriyanghan *et al.*, 2009; Sekhar *et al.*, 2015). In addition to *ETR2*, up-regulation of *ERS1*, *ERS2*, and *ETR3* may also be responsible for poor grain filling of inferior grains (Sekhar *et al.*, 2015). ABA acts as a sensitive signal during drought stress in plants, and its role in grain filling is complicated. The endogenous level of ABA is regulated not only by its biosynthesis, but also by its catabolism. Phaseic acid (PA), one of the primary catabolites of ABA, is catalysed by ABA 8´-hydroxylase (ABA8ox), which contains three cytochrome P450 genes in rice (Saika *et al.*, 2007). It has been reported that cell division and grain-filling rates are significantly and positively correlated with ABA content (Yang *et al.*, 2006*b*). It is notable that ABA plays a key role in grain filling through regulating sink activity, and it functions in a dose-dependent manner (Wang *et al.*, 2015). An appropriate concentration of ABA can enhance the activities of enzymes involved in sucrose cleaving and starch synthesis, and can increase the expression of genes related to starch metabolism (Wang *et al.*, 2015). However, the molecular mechanisms by which hormones regulate grain filling, especially that of inferior grains, under conditions of soil drying remains unclear. Transcription factors (TFs) are essential regulators involved in various biological processes (Shamimuzzaman and Vodkin, 2013), and play crucial roles in plant growth and development, and in response to a range of abiotic stresses (Singh *et al.*, 2002). During the process of grain filling in plants, multiple families of TFs have been observed to be differentially regulated in inferior grains under soil drying conditions. The TF families of AP2 (Oh *et al.*, 2009) and NAC (Sperotto *et al.*, 2009; Uauy *et al.*, 2006) have been reported to participate in regulating the grain-filling process.

With the development of next-generation sequencing platforms, differences during grain filling between superior and inferior grains have been investigated at the omics level with a view to elucidating the mechanisms of grain filling. For instance, sequencing of seeds during different grain-filling stages of superior and inferior grains has identified differentially expressed miRNAs that may help explain poor grain filling in rice (Zhai *et al.*, 2013; Peng *et al.*, 2014). These differently expressed miRNAs have been shown to be involved in plant hormone homeostasis and starch accumulation, suggesting that miRNA-mediated hormonal pathways and starch synthesis might be crucial for grain filling (Zhai *et al.*, 2013; Sun *et al.*, 2015). Proteomic comparisons of inferior grains under water stress and well-watered conditions has shown that soil drying improves the activity of proteins associated with photoassimilate supply and conversion (Dong *et al.*, 2014; Chen *et al.*, 2016). In addition, comparative proteomics of superior and inferior spikelets during early grain-filling stages in ‘super rice’ varieties has shown that endosperm cell division in inferior spikelets is lower than that of superior spikelets as a result of greater evolution of ethylene in inferior spikelets (Das *et al.*, 2016). However, the mechanism of grain filling involves various biochemical processes that have yet to be elucidated. Furthermore, the mechanisms underlying the increase in grain filling in inferior grains that results from moderate water stress and the corresponding increase in inferior grain weight are also unclear. Therefore, in this study, we investigated the expression profiles of genes involved in grain filling in inferior grains under moderate water stress.

## Materials and methods

### Plant material and growth conditions

Field experiments were conducted at the genetic garden of the Chinese University of Hong Kong, China, during the rice growing season (March–August). An inbred rice indica cultivar (*Oryza sativa* subsp. *indica*) YD6 (YD) was grown in growth pools (width 1.5 m, length 5 m) that were filled with paddy soil. The soil water content was monitored by using a tension meter. Seeds were germinated in darkness in Petri dishes with moist filter paper at 28 °C for 2–3 d until the roots measured 1 cm. Germinated seedlings were transferred onto a black mesh that was kept floating on Kimura B nutrient solution and 15-d-old seedlings were planted with one seedling per hill. Fertilizer was according to normal agricultural practice, as described previously by Zhang *et al.*, (2012). The water level in the pools was maintained at 1–2 cm until 9 d after anthesis (DAA), when the moderate soil drying treatment was initiated.

### Water stress treatments

At 9 DAA the application of water to the different pools was adjusted to produce two different treatments. The well-watered control (CK) plants were maintained in a water depth of 1–2 cm (soil water potential equal to 0 kPa) by manually applying tap water, while the plants subjected to moderate drying (MD) were maintained with a soil water potential of –25 kPa. Each treatment had three pools as replicates. The soil water potential in the pools used for the MD treatment was monitored at a soil depth of 15–20 cm using tension meters consisting of a 5-cm long sensor (manufactured by Institute of Soil Science, Chinese Academy of Sciences, Nanjing, China). Four tension meters were installed in each pool to provide an even distribution, and readings were taken twice daily at 10.00 h and 16.00 h. When the readings in a pool dropped to a mean value of –25 kPa, 40 l of tap water was added evenly. The pools were protected from rain by covering them with a polyethylene shelter.

### Sampling

We selected 200 panicles that headed on the same day in each treatment, which were tagged to give an accurate record of the flowering date and the position of the spikelets. Sampling was conducted with a randomized block design with three replicates plots (1×1 m) in each pool. Superior grains that ﬂowered on the first 2 d of anthesis (located on apical primary branches) and inferior grains (located on proximal secondary branches) that flowered on the last 2 d were separated from the panicles (Chen *et al.*, 2013). Thirty tagged panicles from each pool were sampled at 12, 18, and 24 DAA. The sampled panicles were divided into three groups (10 panicles each) as three replicates. Then, superior and inferior spikelets were separated from the panicles for RNA extraction and measurement of soluble sugars, starch, ABA, and enzyme activities (see below). All the sampled grains were dehulled and immersed in liquid nitrogen and then kept in a –80 °C freezer for further analysis. A further 30 tagged panicles (10 panicles formed a subsample) from each treatment were sampled to measure the dry weight of superior and inferior grains at final harvest. The sampled grains were dried at 70 °C to constant weight, dehulled, and weighed.

### Assays of grain weight, soluble carbohydrates, and starch content

To investigate how soil drying affects the grain-filling process, we measured the weight of superior and inferior grains, and the contents of soluble sugars and starch of the inferior grains under the two water supply treatments. A total of 100 superior and inferior grains each were used for measurement of grain dry weight. The samples used for measuring the starch and sucrose contents were ground into fine powder, 500 mg subsamples were transferred to 15-ml centrifuge tubes and 10 ml of 80% ethanol (v/v) was added. The tubes were kept in a water bath at 80 °C for 30 min. After cooling the tubes in water, they were centrifuged at 5000 *g* for 10 min. The supernatant was collected and the extraction was repeated three times. The sugar extract was then diluted to 50 ml with distilled water and the sucrose content was measured as described by Yang *et al.* (2001*a*, 2001*c*). The residues left in the centrifuge tubes after extracting sugars were dried at 80 °C for starch extraction using HClO_4_ following the method described by Yang *et al.* (2001*c*).

### RNA extraction, sequencing, and library construction

Inferior grains sampled at 12, 18, and 24 DAA under the two water supply treatments were used for RNA-sequencing (RNA-seq) analysis. Total RNA was extracted using a RNeasy Plant Mini Kit (Qiagen) followed the method described previously (Wang *et al.*, 2017). Three biological replicates were used. Libraries were constructed according to the method described previously (Wang *et al.*, 2017). The library was then sequenced from both the 5´- and 3´-ends on the paired end using an Illumina HiSeq4000 PE101. The raw image data generated by sequencing were transformed by base-calling into sequence data, called raw data/raw reads, and was stored in fastq format (Liu *et al.*, 2015). Transcriptome data was analysed according to Pertea’s protocol (Pertea *et al.*, 2016). Briefly, clean data (high-quality reads) were mapped to the reference genome (ftp://public.genomics.org.cn/BGI/rice/rise2/9311_genome.fa.gz) using HISAT2 v2.0.5(Kim *et al.*, 2015). The RPKM values (reads per kilo bases per million reads) were calculated using StringTie v1.3.3 (Pertea *et al.*, 2015), followed by differential expression analysis using the Ballgown package in Bioconductor (Frazee *et al.*, 2015). Genes with fold-change (absolute value) >2 and a *P*-value <0.05 were filtered as differentially expressed genes.

### ABA measurement

Endogenous ABA levels of grains were measured using modified methods of Bollmark *et al.* (1988 and He (1993). Samples of stems (three biological replicates) were ground in a mortar at 0 °C in 10 ml of 80% (v/v) methanol extraction medium containing 1 mM butylated hydroxytoluene as an antioxidant. The extract was incubated at 4 °C for 4 h and centrifuged at 4800 *g* for 15 min at 4 °C. The supernatants were sequentially passed through Chromosep C18 columns (C18 Sep-Park Cartridge, Waters Corp, Milford, MA, USA), pre-washed with 10 ml of 100% and 5 ml of 80% methanol. The hormone fractions were dried under N_2_ and dissolved in 2 ml of phosphate-buffered saline (PBS) containing 0.1% (v/v) Tween-20 and 0.1% (w/v) gelatin (pH 7.5) for analysis by ELISA. The mouse monoclonal antigen and antibody against ABA and immunoglobulin G–horseradish peroxidase (IgG–HRP) used in the ELISA were produced at the Phytohormones Research Institute, China Agricultural University (He, 1993). The method for quantification of ABA by ELISA was described previously by Yang *et al.* (2001*c*). ABA concentration was expressed on a fresh weight (FW) basis. The specificity of the monoclonal antibody and the possibility of other non-specific immunoreactive interference had been checked previously (Wu *et al.*, 1988; Yang *et al.*, 2001*c*). HPLC was used to verify the ABA content measured by ELISA (Supplementary Fig. S2 at *JXB* online).

### Enzyme extraction and assays

Proteins were extracted and their content determined by the method of Bradford (1976), using BSA as a standard. The methods for SuSase extraction and measurement of its activity were as described by Ranwala and Miller (1998). Grains were ground with a mortar and pestle in 100 mM HEPES (pH 7.5) containing 10 mM isoascorbate, 3 mM MgCl_2_, 5 ml DTT, 2 ml of EDTA, 5% (v/v) glycerol, 3% (w/v) polyvinylpyrrolidone (PVP), and 0.01% Triton X-100. After centrifugation at 15 000 *g* for 30 min, the supernatant was desalted on a Sephadex G-25 column and the proteins were eluted using a reaction buffer that contained 50 mM HEPES (pH 7.5), 10 mM MgCl_2_, 2 mM EDTA, and 3 mM DTT. The extraction procedures for AGPase, SSS, and SBE were according to Nakamura *et al.* (1989). Briefly, 40–50 grains were ground with a pestle in a pre-cooled mortar that contained 4–8 ml of frozen extraction medium: 100 mM HEPES-NaOH (pH7.6), 8 mM MgCl_2_, 5 mM DTT, 2 mM EDTA, 12.5% (v/v) glycerol, and 5% (w/v) insoluble PVP40. After being filtered through four layers of cheesecloth, the homogenate was centrifuged at 12 000 *g* for 10 min, with the supernatant being used for the enzyme assays. Activities of enzymes are expressed as units mg^–1^ protein min^–1^ for SBE and nmol mg^–1^ protein min^–1^ for the other enzymes.

### *In vivo* analysis of NAC promoter activity on *WAXY*

The 2000-bp sequence of the native *WAXY* promoter (Pro-WAXY) was amplified from genomic DNA. The amplified promoter was cloned into the *pGREENII-0080-luc* vector using a one-step cloning kit (Vazyme, Nanjing, China) to form the reporter construct. The CDS region of the NAC transcription factor was then amplified and cloned into the *pGREENII-62-SK* vector using a one-step cloning kit (Vazyme) to form the effector construct. The two constructed vectors were then mixed well for transient expression assays in the protoplast. The transient expression assays were performed in protoplasts of the rice leaves as described previously by Zhang *et al.* (2011).

### Statistical analyses

Statistical analysis of the data was performed by using ANOVA and Tukey’s *post hoc* test to determine least-significant differences using the software SPSS 19.0 (SPSS Inc., Chicago, IL, USA), and the results are expressed as means (±SD) of three biological replicates. *Post hoc* comparisons to analyse the variable data of each population were conducted using Tukey’s test at *P*<0.05. Correlations were examined using Pearson’s correlation coefficient.

## Results

### Physiological characteristics

**Fig. 1.**
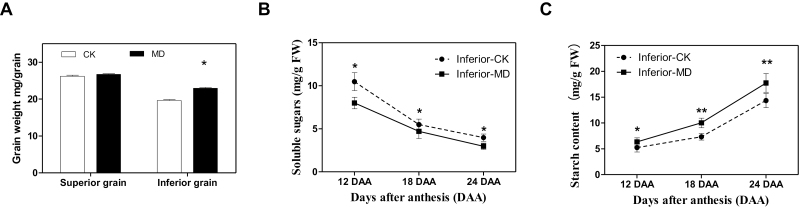
Moderate soil drying applied at post-anthesis alters the contents of soluble sugars and starch in inferior grains of rice during grain filling. (A) Dry weights at harvest of superior and inferior grains subjected either to moderate drying (MD) of the soil or to control (CK) conditions. (B) Content of soluble sugars of the inferior grains at three points during the filling period. (C) Starch content of the inferior grains at three points during the filling period. Values are means (±SD) of three replicates. Significant differences were determined using ANOVA and Tukey’s *post hoc* test: *P<0.05, **P<0.01.

The superior grains, associated with earlier-flowering spikelets, showed no significant difference between the control (CK) and moderate drying (MD) treatments in terms of grain weight (Fig. 1A). However, the weight of inferior grains subjected to MD during grain filling was significantly increased (Fig. 1A). The soluble sugar content of the inferior grains was significantly reduced in the MD treatment (Fig. 1B), whilst the starch content was significantly increased (Fig. 1C).

### Differential expression analysis

Correlations of the expression patterns among the different inferior grain samples are presented in Supplementary Fig. S1A and they show that the biological replication of the six samples (2×treatments, 3×DAA) was appropriately reflected in their respective transcriptomes. The correlation values for the three replicates were all over 0.96. The distribution of RPKM density was used to investigate the gene expression pattern in each sample. Genes showing an intermediate level of expression were highly represented, while those presenting low and high expression levels were the least represented (Supplementary Fig. S1B).

**Fig. 2.**
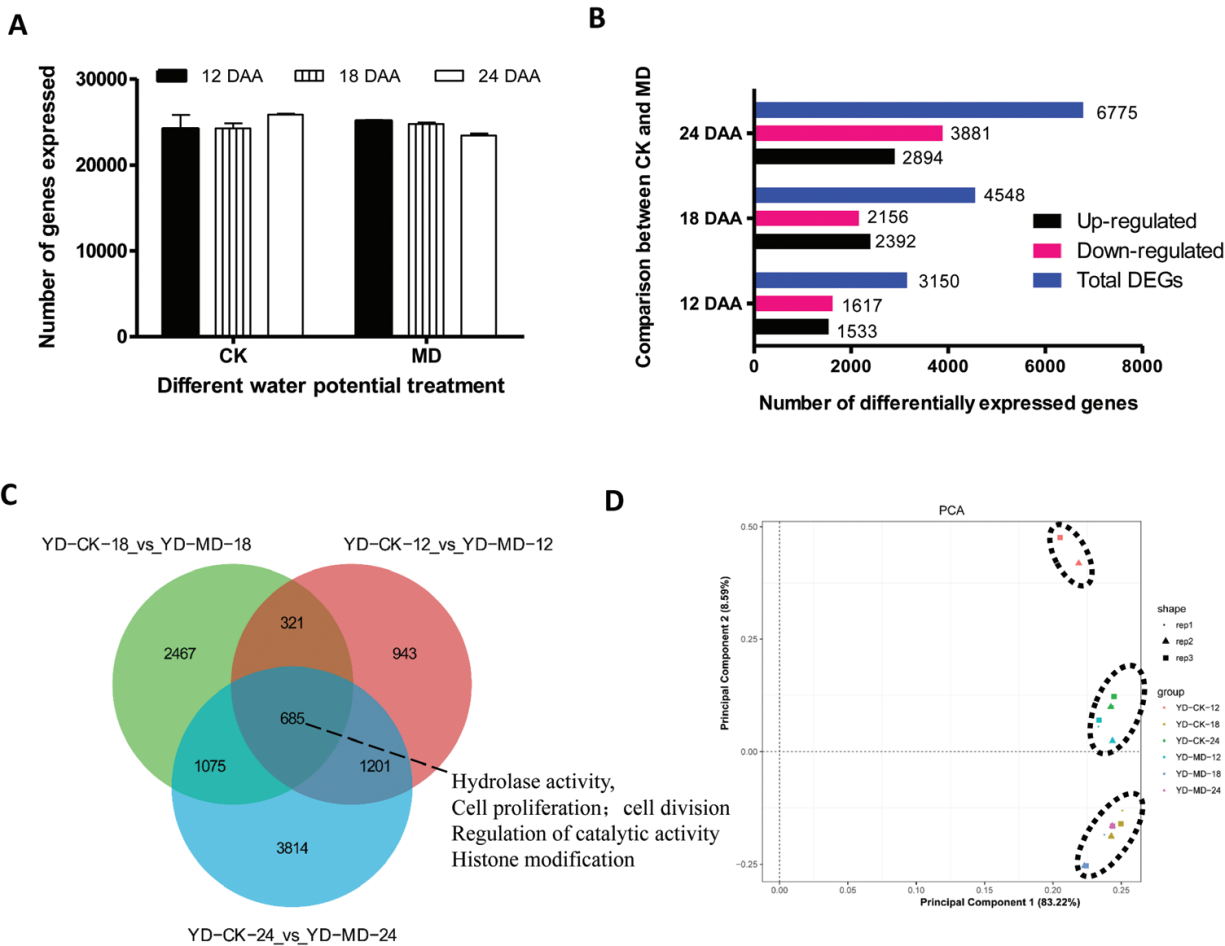
Differential gene expression of inferior grains of rice subjected to moderate soil drying during grain filling. (A) Number of genes detected in each of the samples. (B) Numbers of genes up- and down-regulated and total number of differentially expressed genes (DEGs) in the comparisons between well-watered controls (CK) and moderate drying (MD) conditions at 12 d after anthesis (DAA), 18 DAA, and 24 DAA. (C) Venn diagram showing the DEGs in the three pairwise comparisons between CK and MD conditions at the three time-points during grain filling. (D) Principal component analysis (PCA) based on all the expressed genes. Three distinct groups are clustered based on the gene expression level of each sample.

More than 23 120 genes were expressed in at least one sample (Fig. 2A) and little fluctuation in the total number of genes was found among the six samples. The differentially expressed genes (DEGs) significantly reflected the transcriptional profile corresponding to the difference between the two treatments. We calculated the number of up- and down-regulated genes in each of the pairwise comparisons. The numbers of genes that were up- or down-regulated in the comparisons of CK versus MD at 12 DAA and 18 DAA were similar (Fig. 2B). Approximately 1533 and 1617 genes that were up- or down-regulated, respectively, in the MD treatment compared with the CK treatment were identified at 12 DAA. The numbers of up- or down-regulated DEGs at 18 DAA increased to 2392 and 2156, respectively. At 24 DAA, the number of DEGs increased markedly, with a significant difference between the up- and down-regulated genes. The number of total DEGs gradually increased from 12 DAA to 24 DAA (Fig. 2B). The dataset was also queried to detect DEGs associated with different stages among the various pairwise comparisons. A total of 685 common DEGs were identified in the three comparisons (Fig. 2C). The significantly enriched DEGs (AgriGO, *P*<0.05, FDR<0.05) overlapped among the three stages according to Gene Ontology biological processes and were clustered in ‘hydrolase activity’, ‘cell proliferation’, ‘cell division’, ‘regulation of catalytic activity’ and ‘histone modification’. In addition, 943, 2467, and 3814 DEGs were uniquely up- or down-regulated in the comparisons conducted at 12 DAA, 18 DAA, and 24 DAA, respectively (Fig. 2C). Approximately 321, 1075, and 1201 DEGs were uniquely shared between the pairwise comparisons of 12 DAA versus 18 DAA, 18 DAA versus 24 DAA, and 24 DAA versus 12 DAA, respectively (Fig. 2C).

A principal component analysis (PCA) based on all the genes detected in the six libraries was plotted to confirm the similarity of the transcriptomes between the samples under different treatments (Fig. 2D). The analysis revealed three discrete groups: the transcriptome of CK at 12 DAA formed a single cluster; another group clustered around CK at 24 DAA and the MD treatment at 12 DAA; and the third group consisted of a cluster of CK at 18 DAA and MD at 18 DAA and 24 DAA. The distance between CK and MD was greatest at 12 DAA, followed by that at 24 DAA. The comparison conducted at 18 DAA exhibited the shortest distance (Fig. 2D). Taken together, the PCA results indicated that MD contributed to distinct transcriptomic profiles in inferior grains at 12 DAA and 24 DAA.

### KEGG analysis of DEGs

**Fig. 3.**
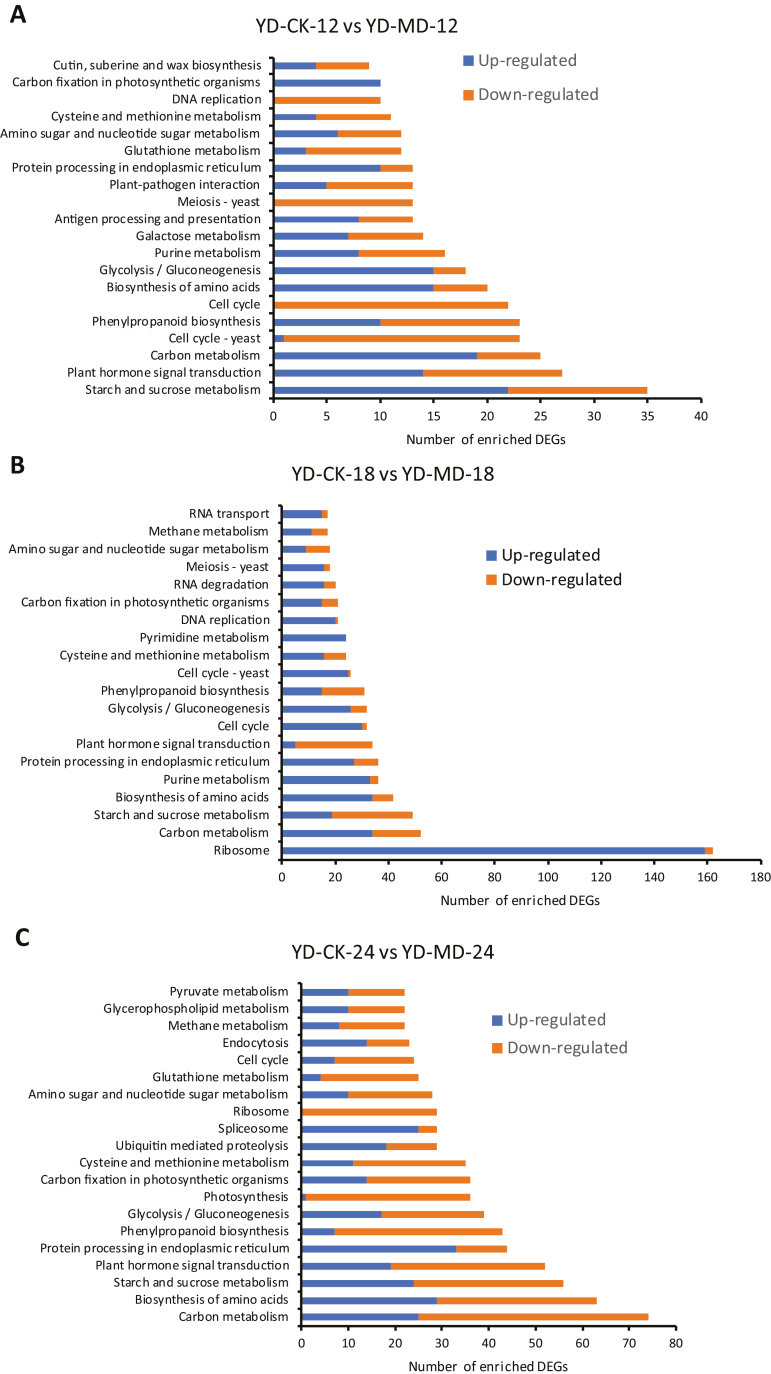
The top 20 enriched KEGG pathways in inferior grains of rice subjected to moderate soil drying during grain filling. The indica rice variety YD6 (YD) was used and plants were either well-watered controls (CK) or subjected to moderate drying (MD) of the soil. Samples were taken at 12 d after anthesis (DAA), 18 DAA, and 24 DAA. The numbers of up- and down-regulated differentially expressed genes (DEGs) enriched for the KEGG pathways of control versus MD are shown at (A) 12 DAA, (B) 18 DAA, and (C) 24 DAA.

The Kyoto Encyclopedia of Genes and Genomes (KEGG, https://www.genome.jp/kegg/) pathway network was used to identify the enrichment of DEGs among different metabolic pathways in the inferior grains. A total of 513 and 410 up- and down-regulated DGs, respectively, were significantly enriched in the transcriptome profile of the MD treatment compared with that of CK at 12 DAA. The significant differences in the top 20 enriched KEGG pathways between CK and MD at 12 DAA are shown in Fig. 3A. The pathway ‘starch and sucrose metabolism’ was the most enriched, followed by ‘plant hormone signal transduction’ and ‘carbon metabolism.’ The numbers of enriched DEGs in these three pathways were 35, 27, and 25, respectively. At 18 DAA, the analysis showed that the pathways ‘ribosome’, ‘carbon metabolism’, and ‘starch and sucrose metabolism’ were enriched with 162, 52, and 49 DEGs, respectively, CK and the MD treatment. In addition, the ‘plant hormone signal transduction’ pathway was enriched with 34 DEGs at this stage (Fig. 3B). At 24 DAA, a total of 975 and 1278 up- and down-regulated DEGs, respectively, were significantly enriched. The top four enriched metabolic pathways were ‘carbon metabolism’, ‘biosynthesis of amino acid’, ‘starch and sucrose metabolism’, and ‘plant hormone signal transduction’ and included 74, 63, 56, and 52 enriched DEGs, respectively (Fig. 3C). The three pairwise comparisons shared the same top pathways in terms of ‘starch and sucrose metabolism’ and ‘plant hormone signal transduction.’

### Expression of genes and activities of enzymes related to starch and sucrose

**Fig. 4.**
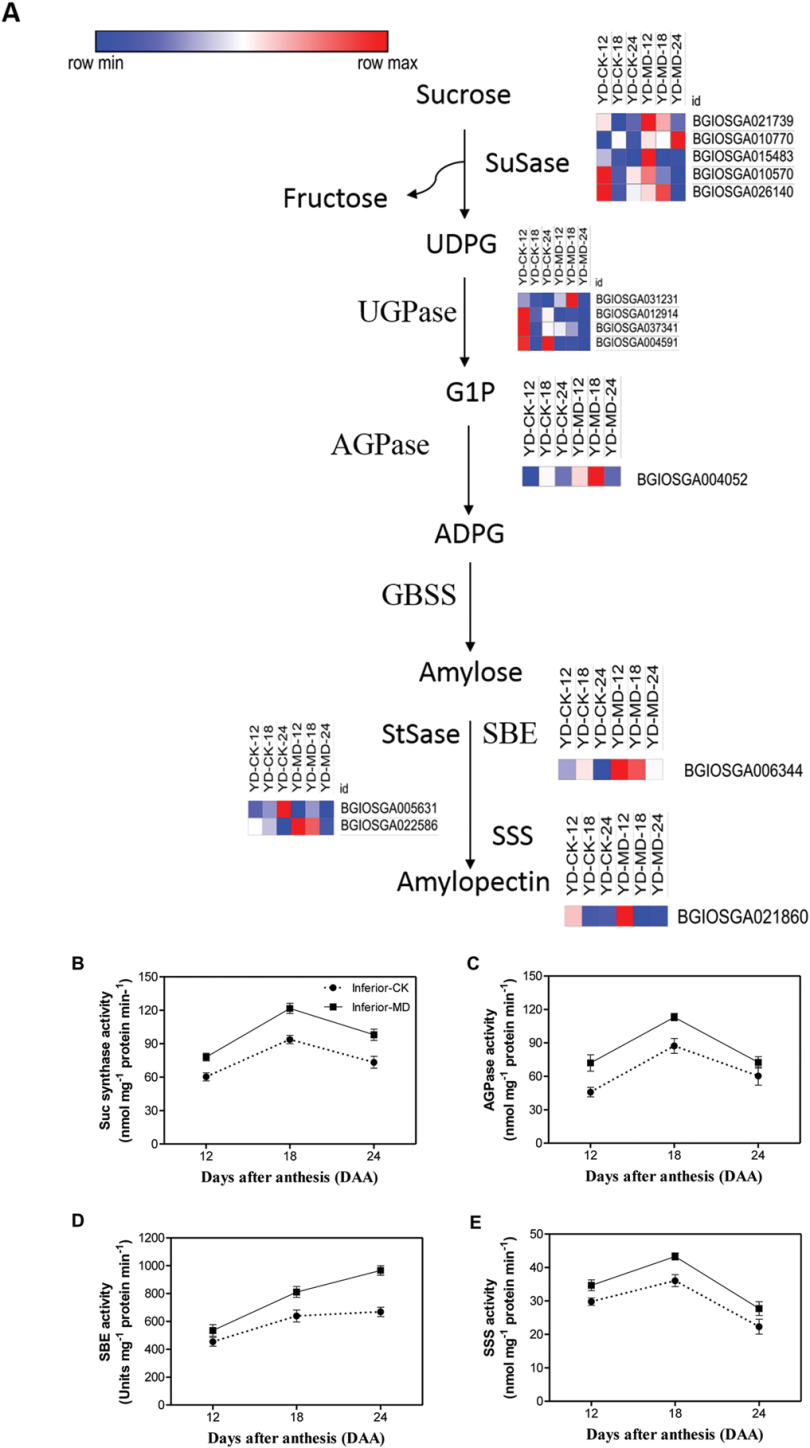
Expression patterns of genes involved in the starch biosynthesis pathway in all transcriptomes and changes in activities of key enzymes in inferior grains of rice subjected to soil drying during grain filling. (A) Heat maps of genes involved in the starch biosynthesis pathway. The maps were plotted using the value of RPKM for each gene in the different samples: blue indicates low values, red indicates high values. The indica rice variety YD6 (YD) was used and plants were either well-watered controls (CK) or subjected to moderate drying (MD) of the soil. Samples were taken at 12 d after anthesis (DAA), 18 DAA, and 24 DAA. (B–E) Enzyme activities of (B) SuSase, (C) AGPase, (D) SBE, and (E) SSS. CK and MD represent well-watered controls and moderate drying (MD) of the soil, respectively. Values are means (±SD) of three replicates.

A heatmap of notable DEGs in the starch biosynthesis pathway in the inferior grains was plotted (Fig. 4A). At 12 DAA, 22 and 13 up- and down-regulated DEGs, respectively, were detected between CK and MD. Among all of the DEGs, genes encoding four key enzymes in the process of converting sucrose into starch were significantly up-regulated in the MD treatment, namely SuSase, AGPase, StSase, and SBE. There were 49 DEGs detected in the pairwise comparison conducted at 18 DAA, including 19 and 30 up- and down-regulated, respectively. The expression of two genes responsible for SuSase were also significantly increased in the MD treatment. Notably, six genes encoding enzymes related to starch degradation were down-regulated under MD conditions, including the beta- and alpha-amylase genes. At 24 DAA, a total of 24 and 32 up- and down-regulated genes, respectively, displayed significant differences between the two treatments. Of the 24 up-regulated DEGs, six genes encoding the four key enzymes SuSase, AGPase, StSase, and SBE were highly expressed under the MD treatment (Fig. 4A). No significant DEGs encoding GBSS, which is responsible for one of the steps in the starch biosynthesis pathway, were identified in these samples, indicating that it might not be involved in the improvement of grain filling induced by soil drying.

We measured the activities of the enzymes involved in the process of sucrose-cleaving and starch synthesis. The activity of SuSase (assayed for cleavage) was higher in the MD treatment than in CK at the three grain-filling stages that we examined (Fig. 4B). The activity increased from 12 DAA and reached a peak at 18 DAA for both the MD CK treatments, but the activity was greatly increased in the MD treatment, which was consistent with the transcription data (Fig. 4A). The enzymes involved in starch synthesis, AGPase, SSS, and SBE, displayed similar patterns of activity to SuSase, although SBE did not show a decline at 24 DAA (Fig. 4C–E). The activities of these enzymes were all increased in the MD treatment at all three measurement times.

### ABA signal transduction

**Fig. 5.**
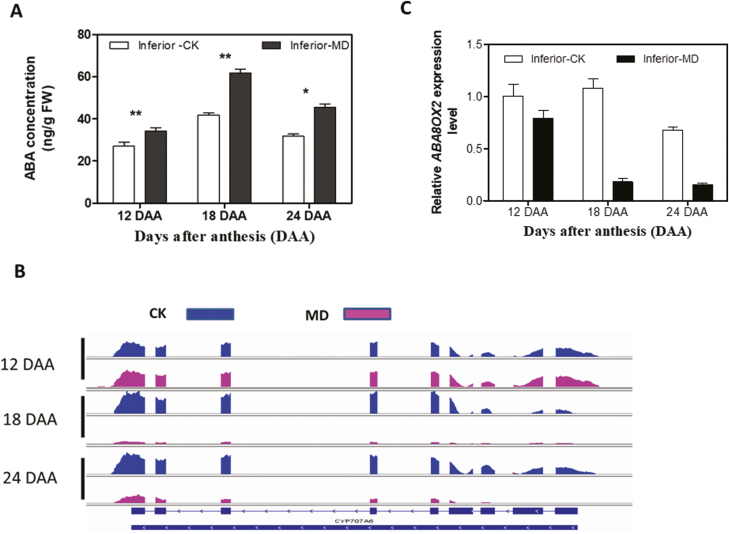
ABA concentrations and expression profiles of an ABA catabolism gene in inferior grains of rice subjected to drying soil during grain filling. (A) ABA levels in grains of well-watered controls (CK) and grains of plants subjected to moderate drying (MD) of the soil. Values are means (±SD) of three replicates. Significant differences were determined using ANOVA and Tukey’s *post hoc* test: *P<0.05, **P<0.01. (B) Differentially expression of *ABA8ox2* (CYP707A6) in inferior grains under CK and MD conditions as visualized using Integrative Genomics Viewer (http://software.broadinstitute.org/software/igv/). (C) *ABA8ox2* expression profiles in inferior grains under CK and MD conditions at three time-points during grain filling. Values are means (±SD) of three replicates.

**Fig. 6.**
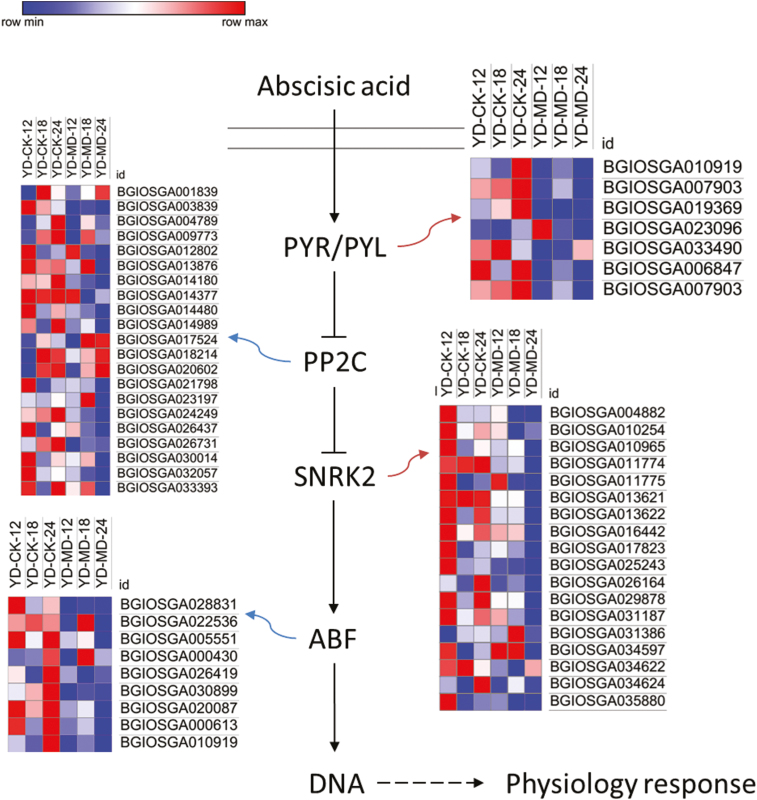
Heat maps of expression data of genes involved in the ABA signal transduction pathway in inferior grains of rice subjected to soil drying during grain filling. The indica rice variety YD6 (YD) was used and plants were either well-watered controls (CK) or subjected to moderate drying (MD) of the soil. Samples were taken at 12 d after anthesis (DAA), 18 DAA, and 24 DAA. The maps were plotted using the value of RPKM for each gene in the different samples: blue indicates low values, red indicates high values

We used ELISA to measure the ABA contents of the inferior grains under the MD and CK treatments at three grain-filling stages (Fig. 5A). The ABA concentration was significantly increased in the MD treatment compared with CK at all the stages, reaching a peak at 18 DAA followed by a reduction at 24 DAA. The pattern of the results using ELISA was consistent with those obtained using HPLC at 24 DAA (Supplementary Fig. S2). Since the endogenous ABA level is regulated by both biosynthesis and catabolism, we investigated the relevant genes in all the libraries. We found that *ABA8ox2* was predominantly detected in our samples (Fig. 5B), while *ABA8ox1* and *ABA8ox3* exhibited much lower transcription levels (data not shown), suggesting that *ABA8ox2* plays a key role in grain filling. The expression levels of *ABA8ox2* were then verified by RT-qPCR (Fig. 5C), which showed the same expression pattern as the RNA-seq results (Fig.5B). The expression level of *ABA8ox2* was down-regulated in the MD treatment compared with CK in the late grain-filling stages (18 DAA and 24 DAA), which was consistent with the relatively high ABA levels detected in the samples (Fig. 5A). We also examined the expression patterns of ABA synthesis genes, and found that both *NCED1* and *NCED3* were detected by RT-qPCR (Supplementary Fig. S3B, C). However, only *NCED1* was detected at high levels in the six transcriptomes, although without any marked difference between the two treatments (Supplementary Fig. S3A). Both *NCED1* and *NCED3* showed lower levels of expression when compared with *ABA8ox2* (data not shown). A heatmap of the genes involved in ABA signal transduction was generated (Fig. 6). Transcripts of the ABA receptor family were highly expressed under CK conditions but were down-regulated under MD conditions, especially at the later stage of grain filling. For PP2C, more than half of the related genes were down-regulated under the MD treatment. The transcript profile for SNRK2 displayed similar trends to the PP2C and PYR/PYL families. Gene expression levels of ABF transcription factors were greatly down-regulated in the MD samples, except for BGIOSGA022536 and BGIOSGA000430, which were up-regulated at 18 DAA under the MD treatment compared with CK (Fig. 6).

### Changes in the expression profile of transcription factors

**Fig. 7.**
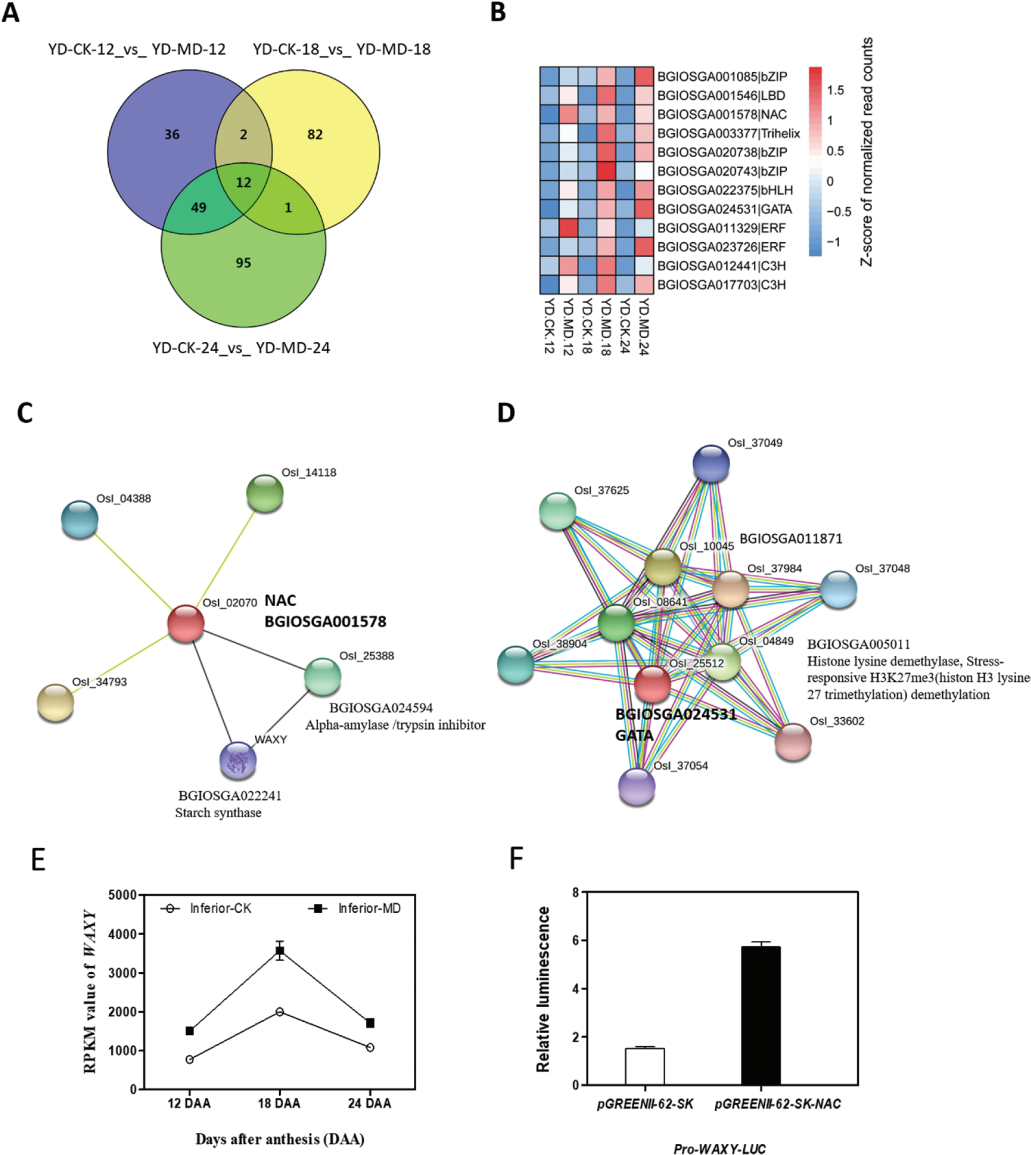
Responses of specific up-regulated transcription factor (TF) families in inferior grains of rice subjected to moderate soil drying during grain filling, and NAC-activated *WAXY* expression. (A) Venn diagram of differentially up-regulated TFs. The indica rice variety YD6 (YD) was used and plants were either well-watered controls (CK) or subjected to moderate drying (MD) of the soil. Samples were taken at 12 d after anthesis (DAA), 18 DAA, and 24 DAA. (B) Heat map of the 12 up-regulated TFs that were common to all the comparisons shown in (A). (C, D) Predicted interacting proteins of NAC and GATA as determined using the Interactions Viewer in STRING (https://string-db.org/cgi/input.pl). (E) Expression profile of *WAXY*, which is involved in starch synthase. Values are means (±SD) of three replicates. (F)Transient expression assay for NAC promoter activity of *WAXY* using luciferase luminescence. Values are means (±SD) of two replicates.

The MD treatment led to a large number of transcription factors (TFs) belonging to multiple families being differentially expressed in inferior grains. In total, 1895 differentially expressed TFs were identified among the three pairs of comparisons. Among these, 201 TF genes were significantly differentially expressed in at least two of the three comparisons (Supplementary Table S1). The number uniquely up-regulated TFs in the three comparisons were 36, 82, and 95 at 12, 18, and 24 DAA, respectively. A total of 12 up-regulated TFs with high expression under MD conditions were common to all three comparisons (Fig. 7A), belonging to eight different families, including ERF, bZIP, GATA, NAC, and C_3_H (Fig. 7B). The BGIOSGA001578 gene encoding a NAC TF was up-regulated under MD conditions and it was predicted to co-express with BGIOSGA024594 (alpha-amylase/trypsin inhibitor) and BGIOSGA022241 (starch synthase) (Fig. 7C), thus indicating the involvement of the NAC TF in grain filling under MD stress. The expression level of BGIOSGA022241 (*WAXY*), which is also involved in starch synthesis, was also up-regulated by MD (Fig. 7E). These findings imply that NAC might possess an activation effect on the transcription of *WAXY*. A transient expression system was then used to investigate whether NAC could activate the expression of *WAXY* in the protoplast. The results showed that co-transformation of a reporter vector with the *WAXY* promoter and an effector vector with *NAC* resulted in significantly higher luciferase activity than co-transformation of the report vector and the control vector without *NAC* (Fig. 7F). In addition, a gene encoding for a GATA factor (BGIOSGA024531) was also shown to have interacting proteins, of which the genes BGIOSGA005011 and BGIOSGA011871 were significantly enriched in the terms of histone demethylation and epigenetic processes (Fig. 7D).

**Fig. 8.**
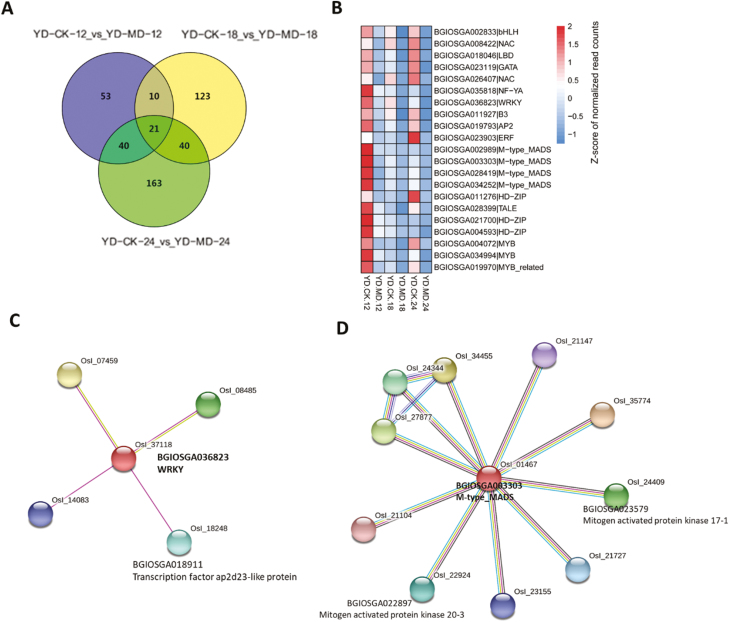
Responses of specific down-regulated transcription factor (TFs) families in inferior grains of rice subjected to moderate soil drying during grain filling. (A) Venn diagram of differentially down-regulated TFs. The indica rice variety YD6 (YD) was used and plants were either well-watered controls (CK) or subjected to moderate drying (MD) of the soil. Samples were taken at 12 d after anthesis (DAA), 18 DAA, and 24 DAA. (B) Heat map of the 21 down-regulated TFs that were common to all the comparisons shown in (A). (C, D) Predicted interacting proteins of WRKY and M-type_MADS as determined using the Interactions Viewer in STRING (https://string-db.org/cgi/input.pl).

**Fig. 9.**
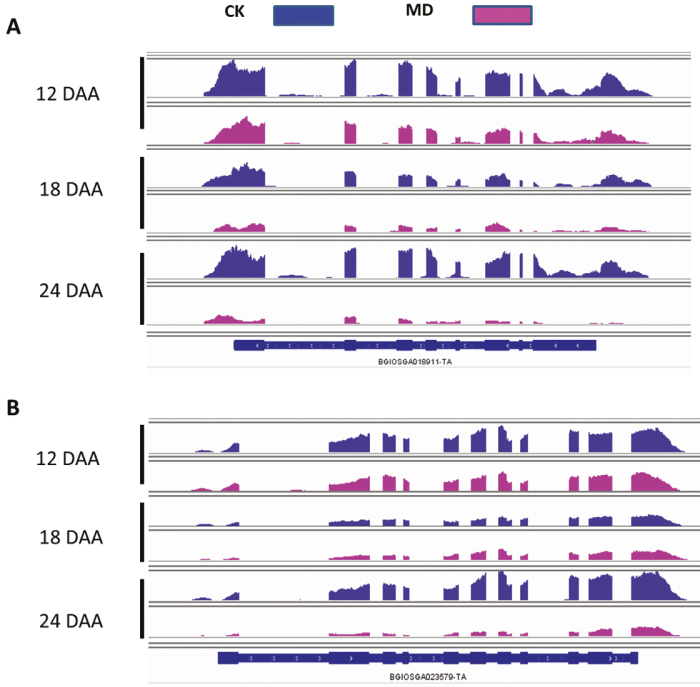
Expression profiles of BGIOSGA018911 and BGIOSGA023579 in inferior grains of rice subjected to moderate soil drying during grain filling as determined using Integrative Genomics Viewer (http://software.broadinstitute.org/software/igv/). The expression profiles of (A) gene BGIOSGA018911 and (B) gene BGIOSGA023579 under well-watered control (CK) and moderate drying (MD) conditions. DAA, days after anthesis.

A total of 21 down-regulated TFs were common to all three comparisons (Fig. 8A), belonging to 13 different families (Fig. 8B). Six of these 21 TFs were predicted to interact with other proteins, namely WRKY, TALE, M-type_MADS, MYB, NF-YA, and GATA. The TF WRKY (BGIOSGA036823) was predicted to interact with BGIOSGA018911, which belongs to the pathway of AP2-like ethylene-responsive TFs (Fig. 8C), suggesting that WRKY might play a significant role in the process of inferior grain filling under soil drying conditions. The expression profile of BGIOSGA018911 also showed down-regulation in response to the MD treatment (Fig. 9A), and showed a similar expression pattern to that of WRKY (Fig. 8B). We also found that M-type_MADS (BGIOSGA003303) was predicted to interact with the mitogen-activated protein kinases 20-3 (BGIOSGA022897) and 17-1 (BGIOSGA023579) (Fig. 8D). Interestingly, the BGIOSGA023579 gene showed a lower expression level under MD (Fig. 9B), and displayed a similar expression pattern of the M-type_MADS (Fig. 8B).

## Discussion

Modern rice varieties that have been developed with large panicles and numerous spikelets have failed to fulfill their high yield potential because of poor grain filling, caused by both a slow filling rate and incompletely filled grains, especially for those that are late flowering (Yang and Zhang, 2006; Sekhar *et al.*, 2015). Abiotic stresses, including drought, have different effects on regulating plant growth. Alternate watering and MD treatments applied at post-anthesis have been shown to increase the grain-filling rate and weight of inferior grains, while only slightly increasing the grain-filling rate of superior grains (Zhang *et al.*, 2012). Continuous MD during the mid-to-late grain-filling stages can increase the grain-filling rate and grain yield of inferior grains (Yang *et al.*, 2000; Yang and Zhang, 2006). In this study, MD applied from 9 DAA until harvest markedly reduced the soluble sugar content and enhanced starch accumulation in inferior grains (Fig. 1B, C). Several studies have focused on the activities of enzymes involved in starch synthesis and the relationship between ABA and filling of inferior grains (Yang *et al.*, 2001*b*, 2003; Zhang *et al.*, 2012). The physiological aspects of the hypothesis that carbohydrates are not a major limiting factor for the development of inferior spikelets has been well studied (Mohapatra *et al.*, 1993; Yang *et al.*, 2006*b*; Zhang *et al.*, 2012). Sink size and sink activity are two factors that influence grain yield. Sink size is generally considered to correspond to the cell number and cell size. Sink activity is more complex, and mainly refers to various biochemical processes including the key enzymes responsible for carbohydrate synthesis and catabolism. Our previous work has shown that MD imposed post-anthesis has no serious effects on the number and size of cells in the rice endosperm (Yang *et al.*, 2000). Therefore, it is reasonable to assume that the positive effects of MD on grain filling and yield of inferior grains are the result of enhanced sink activity; however, the key molecular factors involved remain obscure.

Grain filling is the process whereby carbohydrates are transported from source organs to spikelets. Sucrose is the main substance mobilized from the stems to the grains (Li *et al.*, 2017). When sucrose is translocated to the spikelets, its conversion to starch is initiated, accompanied by the cleavage of sucrose to monosaccharides (Ranwala and Miller, 1998). SuSase is the key enzyme for the first step of sucrose degradation and it is significantly induced by ABA and water stress imposed at the post-anthesis stage. In our study, the genes encoding SuSase were up-regulated in the inferior grains under MD conditions compared with the control (Fig. 4A), which resulted in an increase in the enzyme activity in the inferior grains (Fig. 4B) and led to a significant reduction of soluble sugars (Fig. 1B). The breakdown of sucrose in spikelets resulting from the up-regulation of transcript levels of SuSase genes would further enhance the unloading of sucrose from the phloem to the grains by increasing the gradient between the source and sink (Ranwala and Miller, 1998; Yang *et al.*, 2004). However, although acid invertase also plays a crucial role in sucrose breakdown, its expression was barely detectable under either control or MD conditions (data not shown). Considering all the available evidence, at the transcriptional level, genes encoding SuSase, but not genes encoding acid invertase, are probably crucial for the conversion of sucrose to starch in inferior grains, which is consistent with a previous report that both sucrose synthase and the process of starch filling in inferior grains are affected by ethylene (Naik and Mohapatra, 2000). In addition, the transcription of three other presumably key enzymes, AGPase, StSsase, and SBE, showed higher expression levels in inferior grains under the MD treatment than in the control (Fig. 4A). The up-regulation of expression of these starch biosynthesis-related genes was paralleled by the enhancement of their enzyme activities and starch accumulation during grain filling (Fig. 1C, 4). In addition, the BGIOSGA021860 gene, which encodes the soluble starch synthase 1 (SSS) enzyme, was markedly expressed at 12 DAA under the MD treatment (Fig. 4A), indicating that it might also play key roles in starch synthesis in inferior grains under moderate water stress. Thus, the results of our enzyme assays together with our gene expression analysis demonstrate that the MD treatment acted to regulate enzymes related to the process of sucrose-to-starch conversion in inferior grains during grain filling.

Many studies have demonstrated that hormonal changes at the whole-plant level can regulate senescence and nutrient remobilization (Davies, 2004; Fan *et al.*, 2007). Abiotic stresses also affect hormonal levels, potentially regulating seed development and nutrient mobility (Ober *et al.*, 1991; Andersen *et al.*, 2002). The level of ABA has been demonstrated to be positively correlated with the grain-filling rate in rice under MD (Kato *et al.*, 1993; Kato and Takeda, 1993; Yang *et al.*, 2004; Zhang *et al.*, 2009). It is also presumed that a high concentration of ABA is required to maintain a high grain-filling rate (Kato *et al.*, 1993; Kato and Takeda, 1993; Zhang *et al.*, 2009). In our present study, the ABA concentration was elevated by MD (Fig. 5). Interestingly, the transcripts of ABA synthesis genes were not significantly altered by MD, whereas the *ABA8ox2* gene, which belongs to the ABA catabolism family, was strongly down-regulated by the MD treatment. Since the ABA content was changed by MD during grain filling, we hypothesized that the transcriptional profile of genes involved in the ABA signal transduction pathway in inferior grains might be altered by the MD treatment. The ABA signal transduction pathway consists of four core components, and these proteins form a double-negative regulatory system in which ABA binds to receptors of the PYR/PYL family and forms ternary complexes with clade-A protein phosphatases type 2C (PP2Cs), thereby nullifying their inhibitory effects on SNF1-related protein kinases 2 (SnRK2.2/3/6), and leading to activation of the ABA signaling pathway (Gosti *et al.*, 1999; Fujii *et al.*, 2007; Fujii and Zhu, 2009; Antoni *et al.*, 2012). In our study, lower expression levels of ABA-receptor family genes were observed under MD conditions (Fig. 6). Transcript levels of genes downstream of ABA signal transduction, such as PP2C, SNRK2, and ABF transcription factors were influenced by the MD treatment (Fig. 6). Overall, the elevated ABA content, which improved grain filling, was due to the suppression of *ABA8ox2* expression, rather than the induction of ABA biosynthesis genes by MD. The enhanced ABA levels then altered the expression patterns of genes belonging to the ABA signal transduction pathway, potentially subsequently influencing downstream target gene expression and improving filling in inferior grains under the MD conditions.

We identified a large number of DEGs in the different comparisons made during grain filling (Fig. 2). Multiple families of TFs were observed to be differentially regulated in inferior grains under the MD treatment (Supplementary Table S1). The TF families of AP2 (Oh *et al.*, 2009) and NAC (Uauy *et al.*, 2006; Sperotto *et al.*, 2009) have been reported to participate in regulating the grain-filling process. In our study, NAC was up-regulated by MD (Fig. 7B), and was predicted to be co-expressed with the proteins of starch synthase (WAXY) and an alpha-amylase inhibitor. The transcription level of *WAXY* was also increased by MD (Fig. 7E). Furthermore, a transient expression assay revealed that *NAC* activated the expression of *WAXY* by directly binding to its promoter (Fig. 7F). Our results thus suggested that NAC plays a role in mediating starch synthesis in inferior grains under moderate water stress. The WRKY TF, which was down-regulated, was predicted to interact with the AP2-like ethylene-responsive TF (Fig. 8). In addition, the AP2-like ethylene-responsive TF also displayed lower expression levels under MD conditions (Fig. 9), indicating that fewer AP2 proteins responded to ethylene. The down-regulated M-type_MADS TF was predicted to interact with MAPK (Fig. 8), which plays a role in drought stress (Jonak *et al.*, 1996; Xiong and Yang, 2003; Zhang *et al.*, 2006), indicating that M-type_MADS might also be involved in the response to MD during grain filling in inferior grains. In addition, expression of the GATA factor was differentially regulated by MD in inferior grains (Fig. 7B). This factor was predicted to interact with proteins involved in histone demethylation and epigenetic processes (Fig. 7D), revealing that epigenetic changes might be pivotal in regulating grain filling of inferior grains (Hu *et al.*, 2014; Shi *et al.*, 2014; Yamauchi *et al.*, 2014).

In conclusion, moderate soil drying imposed on rice at the post-anthesis stage enhanced the ABA concentration by down-regulation of *ABA8ox2*, and subsequently altered the expression patterns of genes involved in the ABA signal transduction pathway. Soil drying enhanced the expression of genes encoding enzymes involved in sucrose breakdown and starch biosynthesis, including SuSase, AGPase, StSase, SBE, and SSS, which play crucial roles in the process of starch accumulation in inferior grains. The differential expression that we observed of the transcription factors of NAC, GATA, WRKY, and M-type_MADS, together with related interacting proteins, might be crucial in mediating grain filling of inferior grains under moderate soil-drying conditions. The transcriptional activation that we observed in transient expression assays added weight to the hypothesis that NAC plays a key role of in mediating starch synthesis during grain-filling under soil-drying conditions.

## Supplementary data

Supplementary data are available at *JXB* online.

Fig. S1. Correlations of gene expression patterns and RPKM distribution among the samples.

Fig. S2. ABA content of inferior grains under control and MD treatments at 24 DAA as measured by HPLC.

Fig. S3. Expression profiles of genes involved in ABA synthesis at 12–24 DAA.

Table S1. Differentially expressed transcription factors between the control and moderate soil drying treatments sampled at 12, 18, and 24 DAA.

Supplementary Figures S1-S3

Supplementary Table S1

## Abbreviations

ABA: abscisic acid
AGPase: ADP glucose pyrophosphorylase
DAA: days after anthesis
GBSS: granule-bound starch synthase
MD: moderate drying of soil
NCED: 9-cis-epoxycarotenoid dioxygenase
SBE: starch branching enzyme
SSS: soluble starch synthase
StSase: starch synthase
