# Supplementary material for: Transcriptomic analysis of grain filling in rice inferior grains under moderate soil drying
Source: J Exp Bot. 2019 Jan 23;70(5):1597–611. doi: 10.1093/jxb/erz010 (PMC6411378; doi:10.1093/jxb/erz010)
Supplement: Supplementary Figures S1-S3 [file erz010_suppl_supplementary_figures_s1-s3.pdf]

Regulation of gene expression of carbohydrate accumulation in inferior grains in response to moderate soil drying during grain filling

Guan-Qun Wang[1,2,], Hao-Xuan Li[2,*], Lei Feng[3], Mo-Xian Chen[4], Shuan Meng[1], Neng-Hui Ye[1,2,#] and Jian-Hua Zhang[2,#]

Supplementary Figures

Fig. S1: The correlation of gene expression pattern and RPKM distribution among the samples.

Fig. S2: ABA content of inferior grains under CK and MD treatments at 24 DAA measured by HPLC

Fig. S3: Expression profiles of genes involved in ABA synthesis.

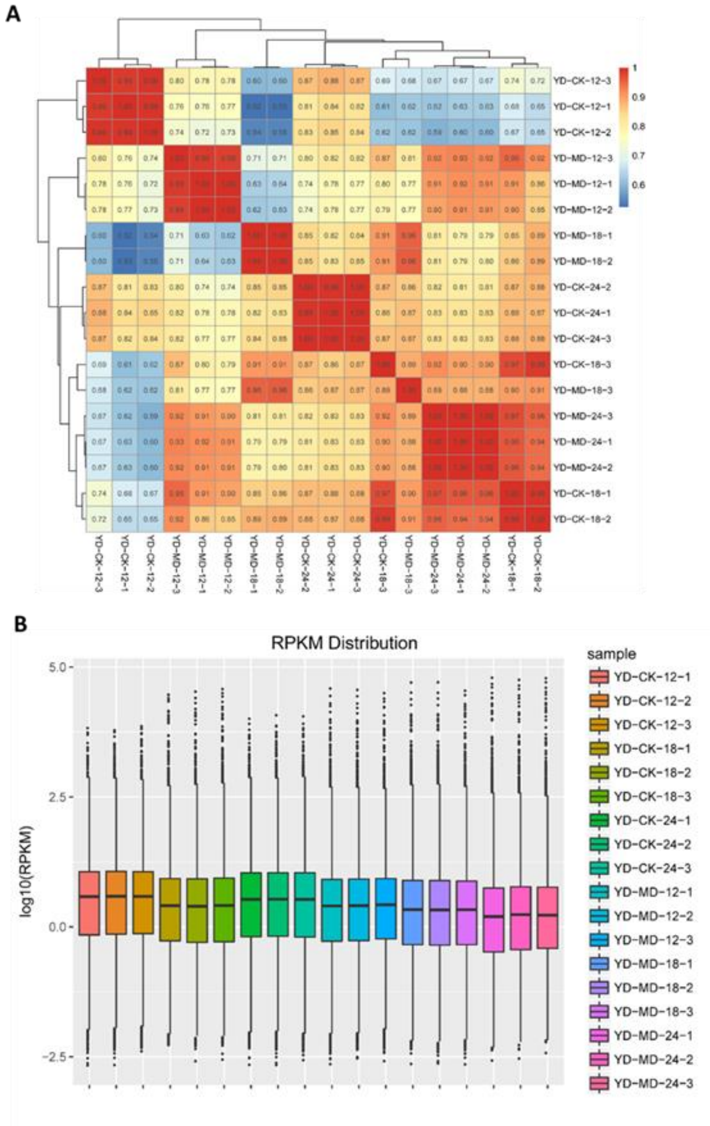

Supplementary Fig. S1 The correlation of gene expression pattern and RPKM distribution among the samples. A, the Pearson's correlations among the samples. The number in the box approximal to 1 means the similarity degree is high. The red color represents the high correlation, the blue color refers to the low correlation. B, The RPKM distribution density among all the samples with three replications.

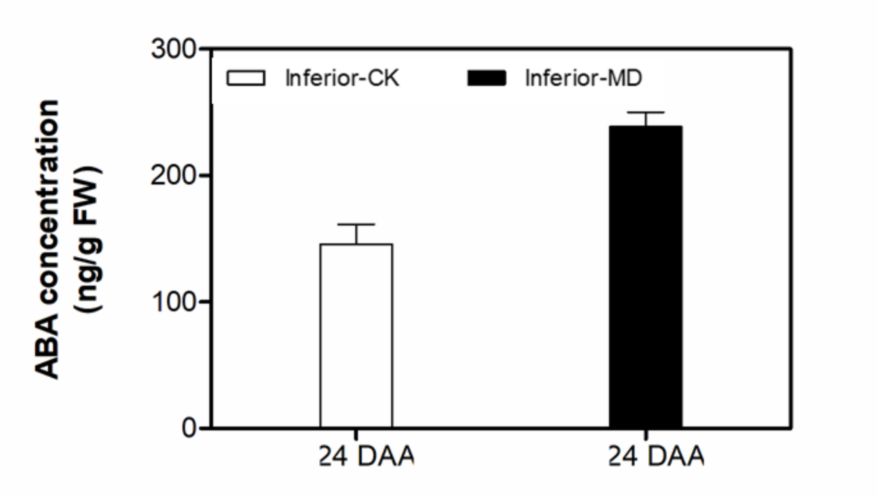

Supplementary Fig. S2 ABA content of inferior grains under CK and MD treatments at 24 DAA measured by HPLC. Error bars represent the means ± SD of three replications.

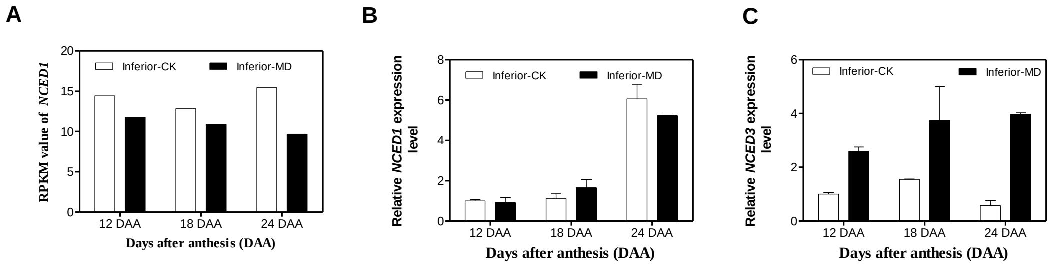

Supplementary Fig. S3 Expression profiles of genes involved in ABA synthesis. A, RPKM value of NCED1 measured by transcriptome. B and C are the expression levels of NCED1 and NCED3 detected by the RT-qPCR results at three grain filling stages. Error bars represent the means ± SD of three replications.
